# Safety of early Hartmann reversal during adjuvant chemotherapy in colorectal cancer: a pilot study

**DOI:** 10.3389/fsurg.2023.1243125

**Published:** 2023-09-27

**Authors:** Dong Ha Kim, Kyung-Ha Lee

**Affiliations:** Department of Colorectal Surgery, Chungnam National University College of Medicine and Hospital, Daejeon, Republic of Korea

**Keywords:** Hartmann procedure, colorectal cancer, chemotherapy, Hartmann reversal, stoma

## Abstract

**Introduction:**

Most patients undergoing the Hartmann procedure for complicated colorectal cancer require chemotherapy because of their advanced status. Stoma created during the procedure is typically closed after the completion of postoperative chemotherapy. However, stomas can induce medical or surgical complications and disturb quality of life. This study aimed to evaluate the safety of Hartmann's reversal during postoperative chemotherapy.

**Methods:**

We conducted a retrospective review of electronic medical records. Between 2017 and 2021, 96 patients underwent Hartmann reversal for after colorectal cancer surgery. Among them, the number of patients who underwent Hartmann procedure with radical resection of complicated colorectal cancer and Hartmann reversal during adjuvant chemotherapy was 13. The clinical, surgical, and pathological characteristics of the patients were evaluated.

**Results:**

Eight and five patients had obstructions and perforations, respectively. Two patients with synchronous liver metastases underwent simultaneous liver resection and reversal simultaneously. Five and eight patients received adjuvant chemotherapy with capecitabine and FOLFOX, respectively. The median interval between the Hartmann procedure and reversal was 3.31 months (2.69–5.59). The median operative time for Hartmann's reversal was 190 min (100–335). The median hospital stay was 10 days (7–21). Four patients (30.8%) developed postoperative complications, and the rate of 3 or higher grade according to the Clavien-Dindo classification within 90 days postoperatively was 0%. Except for 1 patient who refused continuation of chemotherapy, 12 patients completed the planned chemotherapy. Median total duration of adjuvant chemotherapy was 6.78 months (5.98–8.48). There was no mortality.

**Conclusion:**

Early Hartmann reversal during adjuvant chemotherapy is tolerable and safe in carefully selected patients. In particular, it can be used as a therapeutic option for patients with complicated colorectal cancer with synchronous resectable metastases.

## 1. Introduction

Colorectal cancer (CRC) is one of the most commonly diagnosed cancers worldwide and a significant cause of morbidity and mortality (1). Guidelines recommend adjuvant chemotherapy after curative resection in patients with high-risk stage II disease with poor prognostic factors and stage III disease. Poor prognostic factors include pathologic features such as lymphovascular invasion, perineural invasion, tumor budding, and clinical conditions including obstruction and perforation. Approximately 30% of CRCs present as an emergency, of which obstruction accounts for nearly 80%, and perforation for the remaining 20% (2). The most common site of obstruction is the sigmoid colon (3). Emergency surgery for complicated CRCs has a relatively high morbidity and mortality rate, (4) and oncologic outcomes are considered poor compared to elective surgery for uncomplicated CRCs (5). In particular, for left colon and rectal cancer requiring colocolic or colorectal anastomosis, primary anastomosis is considered more difficult and risky than for right colon cancer requiring ileocolic anastomosis. Therefore, many patients require the Hartmann procedure (HP) and, consequently the Hartmann reversal (HR). Hartmann's reversal is a challenging procedure with technical difficulties, and is associated with high morbidity (6). However, there is no recommendation for the appropriate timing of the HR.

Most patients undergoing HR for complicated CRC require chemotherapy because they are usually advanced, and obstruction and perforation are risk factors for poor prognosis. Generally, HR tends to be performed after the completion of chemotherapy, if possible. However, ostomy procedures can cause medical or surgical complications and affect quality of life. However, studies on the safety of HR during chemotherapy are lacking. The aim of this study was to evaluate the safety of the HR during postoperative chemotherapy.

## 2. Methods

This was a single-center, retrospective, observational study.

### 2.1. Patients

Patients who underwent HR during postoperative chemotherapy between 2016 and 2022 were enrolled after reviewing their electronic medical charts. The inclusion criteria were as follows: patients who underwent HP with radical resection of initially diagnosed primary CRC, including those who had HP performed in another institute; patients with CRC indicated for adjuvant chemotherapy (high-risk stage II, stage III, and stage IV with resectable metastases); and those who underwent HR during chemotherapy. The exclusion criteria were as follows: HP for CRC with unresectable metastases, HP for recurrent CRC after initial resection, and double-barrel stoma without the need for laparotomy for stoma closure. This study was approved by the institutional review board (approval number: 2022-07-070).

### 2.2. Staging

For the initial workup, laboratory tests, including carcinoembryonic antigen (CEA) levels, simple radiography of the chest and abdomen, and abdominopelvic computed tomography (CT), were performed. If distant metastasis was suspected, further evaluation was performed after emergency HR. Positron emission tomography was performed in all patients with suspected distant metastases, and liver magnetic resonance imaging and chest computed tomography were respectively performed when liver and lung metastases were suspected.

### 2.3. Surgery

For HP, radical resection with D3 dissection and complete mesocolic excision or tumor-specific total mesorectal excision were performed using the open or laparoscopic method. Division of the distal and proximal ends was performed using a stapler with curved and linear cutters, respectively. Splenic flexure mobilization was only performed partially when it was mandatory for lengthening of the proximal colon to create a colostomy to prevent additional adhesions considering the HR. Appropriate irrigation was performed, and a closed suction drain for obstruction and perforation without severe contamination or a sump drain for perforation with fecal peritonitis was kept in the pelvic and other dependent positions, if necessary. Fifteen grams of the viscous solution adhesion barrier was spread throughout the abdominal cavity, especially around the colostomy site and beneath the main wound. An end colostomy was performed on the upper or lower parts of the left side of the abdomen. The main wound was closed layer-by-layer or in one layer if there was severe bowel dilatation or contamination. In the absence of severe peritonitis, a closed suction drain was clamped for 24 h postoperatively to maximize the efficacy of the adhesion barrier.

A day before HR, administration of oral antibiotic (rifaximin 400 mg and metronidazole 500 mg twice a day), mechanical bowel preparation (polyethylene glycol) and rectal enema were performed. Prophylactic antibiotics was administered within 1 h of incision, postoperatively, and on the morning of postoperative day 1 and discontinued. Hartmann reversal was performed using the open method. After adhesiolysis and removal of the end colostomy, end-to-end anastomosis was performed using a circular stapler with intermittent reinforcement sutures. Full splenic flexure mobilization was performed if necessary. Fifteen grams of the viscous adhesion barrier was spread throughout the abdominal cavity, and the main and colostomy wounds were closed layer-by-layer.

### 2.4. Chemotherapy and examination

After HP, pathological staging was performed by a specialized pathologist according to the 8th American Joint Committee on Cancer (AJCC) TNM system. For stage II and III CRC, adjuvant chemotherapy with FOLFOX or capecitabine was administered for 6 months. The regimens were determined according to the patient's stage, age, and performance status. For stage IV patients with resectable metastases, perioperative chemotherapy without a target agent was administered for 6 months with FOLFOX, and radical resection for metastases was planned with HR.

For stage II and III CRC, examinations, including laboratory tests for CEA levels, chest radiography or CT, and abdominopelvic CT, were performed after 4th cycle of chemotherapy and the completion of chemotherapy. For radically resectable stage IV CRC, examinations were performed after the 4th cycle, 8th cycle and completion of chemotherapy. After completion of chemotherapy, surveillance was performed every 4 months for 2 years and every 6 months for the next 3 years.

### 2.5. Statistical analysis

Statistical analyses were performed using the IBM SPSS Statistics, Version 22 (IBM Corp., Armonk, NY, USA).

## 3. Results

During the 7-year study period, a total of 49 patients underwent HR, and 27 patients among these underwent HP due to complicated colorectal cancer. Among these, 7 patients did not receive chemotherapy due to reasons of age, general condition, or refusal. One patient underwent HR before the initiation of chemotherapy and 6 patients underwent HR after the completion of chemotherapy. These 6 patients underwent late HR due to concern of local recurrence at the rectal stump (*N* = 1) and systemic metastases with priority of chemotherapy (*N* = 5). Consequently, a total of 13 patients were enrolled, and the rate of patients who underwent early HR during chemotherapy (*N* = 13) compared to all patients who underwent HR and chemotherapy (*N* = 20) was 65%.

The clinical and surgical characteristics of the HP are presented in Table 1. Eight and five patients had obstructions and perforations, respectively. Two patients exhibited synchronous liver metastases. Patients underwent upfront HP considering staged liver resection, and further evaluations with liver MRI and PET were performed postoperatively to confirm resectability. Four patients underwent elective HP because of their stable status without severe symptoms. One patient underwent laparoscopic HP surgery. She was initially scheduled to undergo elective laparoscopic low anterior resection (LAR); however, severe colonic dilatation occurred after mechanical bowel preparation, and the operative plan was changed to HP intraoperatively. Two patients developed postoperative ileus and surgical site infections that improved with conservative management.

**Table 1:** Clinical and surgical characteristics of Hartmann procedure.

Factors	Number (%)
Sex	
Male Female	6 (46.2%) 7 (53.8%)
Age (years, mean)	50–80 (65.1)
ASA score	
0,1 2,3	5 (38.5%) 8 (61.5%)
Diagnosis	
Descending colon cancer Sigmoid colon cancer Rectosigmoid colon cancer	1 (7.7%) 8 (61.5%) 4 (30.8%)
Complication of tumor	
Obstruction Perforation	8 (61.5%) 5 (38.5%)
cT	
cT3 cT4	11 (84.6%) 2 (15.4%)
cN	
cN0 cN1 cN2	5 (38.5%) 7 (53.8%) 1 (7.7%)
cM	
cM0 cM1	11 (84.6%) 2 (15.4%)
Emergency	
No Yes	4 (30.8%) 9 (69.2%)
Operative method	
Open Laparoscopic	12 (92.3%) 1 (7.7%)
Operative purpose	
Radical Staged	11 (84.6%) 2 (15.4 5)
Postoperative complication	
No Yes Postoperative ileus Surgical site infection Grade 3 or more Clavien-Dindo classification	11 2 (32.4%) 1 (5.9%) 1 (8.8%) 0 (0.0%)

The pathological characteristics and adjuvant chemotherapy regimens are presented in Table 2. The numbers of patients with stages II, III, and IV were six, five, and two, respectively. Five and eight patients received adjuvant chemotherapy with capecitabine and FOLFOX, respectively. Nine patients experienced adverse effects from chemotherapy.

**Table 2:** Pathologic characteristics and regimen of adjuvant chemotherapy.

Factors	Number (%)
Pathologic T stage	
pT3 pT4	10 (76.9%) 3 (23.1%)
Pathologic N stage	
pN0 pN1 pN2	7 (53.8%) 4 (30.8%) 2 (15.4%)
AJCC stage	
II III IV	6 (46.1%) 5 (38.5%) 2 (15.4%)
Number of retrieved lymph node (Mean)	14–45 (22.31)
Number of metastatic lymph node (Mean)	0–9 (1.62)
Lymphovascular invasion	
No Yes	1 (7.7%) 12 (92.3%)
Perineural invasion	
No Yes	5 (38.5%) 8 (61.5%)
Extramural venous invasion	
No Yes	8 (61.5%) 5 (38.5%)
Regimen of postoperative chemotherapy	
Capecitabine FOLFOX	5 (38.5%) 8 (61.5%)

The surgical characteristics of Hartmann's reversal and hospital course are presented in Table 3. The median interval from HP to HR was 3.31 months (2.69–5.59 months). The Hartmann reversal was planned to be performed 2 months postoperatively, that is, after the 2nd cycle of capecitabine and after the 4th cycle of FOLFOX. As a result, four, one, three, and five patients underwent HR after the 2nd and 3rd cycle of capecitabine and the 4th and 5th cycle of FOLFOX, respectively. Patients who underwent laparoscopic HP also underwent laparoscopic HR. Two patients underwent liver resection (one underwent left lateral sectionectomy, and the other underwent left lateral sectionectomy, S8 tumorectomy, and S6 tumorectomy) with HR simultaneously. The median operative time for HR was 190 min (100–335 min). The median hospital stay was 10 days (7–21 days). Four patients (30.8%) developed postoperative complications. Two of these patients (15.4%) had incisional hernias and underwent herniorrhaphy after completion of adjuvant chemotherapy. Two patients had surgical site infections and C. difficile-induced colitis, which improved with conservative management. Therefore, the rate of 3 or higher grade according to the Clavien-Dindo classification within 30 days and between 30 and 90 days postoperatively was 0%. During adjuvant chemotherapy, the incidences of hand-foot syndrome, fatigue, anorexia, and nausea/vomiting were 30.8%, 30.8%, 7.7%, and 38.5%, respectively. There were no grade 3 or higher adverse events according to (Common Terminology Criteria for Adverse Events). Although 1 patient refused restart of chemotherapy, 12 patients completed the scheduled chemotherapy with the rate of dose reduction of 53.8% (*N* = 7). The median duration of total adjuvant chemotherapy, including HR was 6.78 months (5.98–8.48 months). There was no mortality.

**Table 3:** Surgical characteristic of hartmann reversal and hospital course.

Factors	Number (%)
Time from HP to HR (months, median)	2.69–5.59 (3.31)
Timing of HR	
After #2 Capecitabine After #3 Capecitabine After #4 FOLFOX After #5 FOLFOX	4 (30.8%) 1 (7.7%) 3 (23.1%) 5 (38.5%)
Operative method	
Open Laparoscopic	12 (92.3%) 1 (7.7%)
Combined resection with HR	
None Liver resection	11 (76.9%) 2 (15.4%)
Operation time for HR (minutes, median)	100–335 (190)
Postoperative complication after stoma closure	
None Yes Incisional hernia Surgical site infection Clostridium difficile induced colitis Grade 3 or more Clavien-Dindo classification*	9 69.2%) 4 (30.8%) 2 (15.4%) 1 (7.7%) 1 (7.7%) 2 (15.4%)
Hospital stay for HR (Day, median)	7–21 (10)
Adverse events of chemotherapy	
No Yes Hand-foot syndrome Fatigue Anorexia Nausea/vomiting Grade 3 or more adverse events according to CTCEA Discontinuation^†^ Dose reduction	4 (30.8%) 9 (69.2%) 4 (30.8%) 4 (30.8%) 1 (7.7%) 5 (38.5%) 0 (0.0%) 1 (7.7%) 7 (53.8%)
Duration of adjuvant chemotherapy (*N* = 12^‡^, median)	5.98–8.48 (6.78)

* Incisional hernia which without any complication was found in 2 patients after completion of chemotherapy, and treated with elective herniorrhaphy.

^†^ Discontinuation due to patient refusal without grade 3 or more adverse events.

^‡^ Exception of 1 patient who refused completion of chemotherapy as above mentioned.

## 4. Discussion

Previous studies have consistently reported on the safety of loop ileostomy reversal (7, 8). However, there is a lack of research regarding the safety of early HR.

Hartmann's reversal has conventionally been considered technically challenging and is associated with high morbidity and mortality. In addition, there is no standard guidelines regarding the optimal timing for HR after HP. Several studies have reported conflicting results regarding the appropriate timing. Pearce et al. reported an increased risk of postoperative complications, including anastomotic leakage and mortality, in patients who underwent HR within 6 months of HP compared to those who underwent HR after 6 months (9). Similar results have been reported previously (10). However, Roe et al. reported poor outcomes in patients who underwent HR 4 months after HP compared to those who underwent HR within 4 months, suggesting that early HR should be considered when possible (11). Based on several review articles, the most frequently reported time interval for HR was between 6 and 12 months (12, 13). In 2020, Rasslan et al. reported a median time from HP to HR of 20 months (14). Similarly, in 2021, Bitran et al. reported a median time of 12 months, with only 12% of patients undergoing HR within 6 months (15). More recently, Clementi et al. reported a median time of approximately six months and found that patients who underwent early HR had a significantly lower complication rate than those who underwent late HR (16). Particularly, for patients who undergo HP due to complicated CRC, HR tends to be delayed after the completion of chemotherapy or may not be performed in patients with poor performance or advanced CRC. Horesh et al. reported that patients who underwent HP for malignant conditions had a significantly lower rate of HR than those who underwent HP for benign disease owing to their baseline condition, need for cancer treatment, and worse prognosis (17).

In this study, the median time from HP to HR was 3.31 months, with HR typically performed two months after HP. The timing was determined after the confirmation of the first follow-up CT scan after the second cycle of capecitabine, which was administered triweekly, and after the fourth cycle of FOLFOX, which was administered biweekly. A resting period of at least 2 weeks from the last chemotherapy session to the major operation is generally considered necessary to reduce postoperative complications related to chemotoxicity. At the same time, the next chemotherapy session should not be significantly delayed to ensure oncologic safety. Therefore, if HR was scheduled more than 3 weeks after the last chemotherapy session due to surgeon or patient scheduling constraints, the next chemotherapy session, which included the third cycle of capecitabine and the fifth cycle of FOLFOX, was administered prior to HR. The median operative time was 190 min, which is similar to that reported in previous studies (13). This included the time for liver resection in 2 cases. The challenging and time-consuming nature of HR is typically attributed to the extensive adhesions following septic abdominal conditions. To reduce postoperative adhesions, the antiadhesive agent (Guardix-SG, Genewel, Dongsung Company, Seongnam, Korea) was routinely applied before abdominal wall closure of HP and drained 24 h later. The degree of adhesion during HR varied; however, all adhesions were easily separated. Kim et al. reported a significant reduction in the rate of postoperative obstruction after laparotomy using this agent (18). No case of postoperative obstruction was observed. There were two cases of postoperative complications of grade 3 or higher according to the Clavien-Dindo classification, both of which were incisional hernias that were repaired by elective herniorrhaphy. There was no immediate postoperative complications requiring any intervention or surgery. Surgical site infection is commonly reported after HR (19); however, it occurred in only one case in this study. This suggests that appropriate bowel and skin preparation along with meticulous and aseptic surgical techniques can effectively prevent such infections. The median hospital stay was 10 days, which is consistent with previous reports (13). The application of the Enhanced Recovery After Surgery protocol in both open and laparoscopic surgery contributed to a shorter hospital stay for patients in this study than in the past. In addition, there were no grade 3 or higher adverse events related to chemotherapy after HR, according to CTCEA. The median duration of adjuvant chemotherapy was 6.78 months. Although this was a retrospective study with a small number of patients, the results suggest that HR can be safely performed during adjuvant chemotherapy without significantly prolonging its duration.

The two patients in this study underwent HR with concomitant liver resection. Among the patients who required emergent HP, some presented with distant metastases, mostly in the liver. Most international guidelines recommend upfront liver resection for liver metastases with good technical resectability and favorable prognostic criteria, preoperative chemotherapy followed by radical resection for liver metastases with unfavorable prognostic criteria regardless of technical resectability, and conversion chemotherapy for borderline resectable liver metastases, followed by another MDT discussion after two or three months (20). However, simultaneous liver and CRC resection is difficult in patients requiring emergent HP. Liver resection and prolonged operative time may increase morbidity and mortality in patients with sepsis. In addition, even if liver metastases are considered operable based on CT findings, liver MR is necessary to detect occult metastases that may not be visible on CT scans. Therefore, liver resection with HR after HP and 2 months of chemotherapy may be an ideal treatment option for complicated CRC with liver metastases. However, systemic control with chemotherapy or close surveillance without anastomosis would precede early HR when metastases are currently inoperable or local recurrence is highly suspected. In this study, there were 6 patients who underwent late HR and were excluded from the study. They underwent chemotherapy for more than 6 months without HR to prioritize disease control without delaying chemotherapy due to surgery with high morbidity and mortality. Therefore, patient selection should be done carefully to improve oncologic outcomes.

Limitations of this study include its retrospective nature and small number of patients. Furthermore, this treatment strategy may not be appropriate for patients with general conditions or those who are not candidate for a sequential major surgery. Also, as mentioned above, continuous systemic control without delay or discontinuation may take precedence in patients with advanced disease. Therefore, careful patient selection is critical when considering this treatment strategy. In addition, because the specific protocol of surgery, including antiadhesive material and postoperative management, are not standardized and varies according to the surgeon's preference, it may also be a limitation to widespread application of this strategy based on the experience of a single institution.

In conclusion, based on our findings, early HR during adjuvant chemotherapy is well tolerated and safe in carefully selected patients. This approach may serve as a viable therapeutic option, particularly in patients with complicated CRC and synchronous resectable metastases. However, it is important to note that patient selection is critical when considering early HR. Factors, such as general condition, age, and stage of disease, should be carefully evaluated to ensure that patients are suitable candidates for sequential major surgery. Further research and larger studies are warranted to validate these findings and to establish more comprehensive guidelines for optimal timing of HR in this patient population.

## Data Availability

The raw data supporting the conclusions of this article will be made available by the authors, without undue reservation.
